# Adjunctive Platelet-Rich Plasma (PRP) in Infrabony Regenerative Treatment: A Systematic Review and RCT's Meta-Analysis

**DOI:** 10.1155/2018/9594235

**Published:** 2018-03-19

**Authors:** Mubashir Saleem, Flavio Pisani, Faisal Maqbool Zahid, Ioannis Georgakopoulos, Teuta Pustina-Krasniqi, Edit Xhajanka, Maher Almasri

**Affiliations:** ^1^Faculty of Dentistry, School of Health, MClinDent in Periodontology, BPP University Birmingham, Birmingham, UK; ^2^Department of Statistics, Ludwig-Maximilians-University Munich, Munich, Germany; ^3^World Academy of Growth Factors and Stem Cells in Dentistry, Athens, Greece; ^4^Universiteti I Prishtines “Hasan Prishtina”, Prishtine, Kosovo; ^5^Universiteti I Mjekesise Dentare, Tirane, Albania

## Abstract

**Background and Objective:**

The purpose of this study was to highlight the clinical performance of platelet-rich plasma (PRP) used as an adjunctive tool for regeneration in infrabony periodontal defects using different biomaterials or performing different surgical flap approaches. Comparative evaluation of main clinical outcomes as probing pocket depth reduction, clinical attachment gain, and recession reduction with and without the use of PRP has been analysed.

**Materials and Methods:**

According to the focused question, an electronic and hand searching has been performed up to December 2016. From a batch of 73 articles, the selection strategy and Jadad quality assessment led us to include 15 studies for the meta-analysis.

**Results:**

Despite the high heterogeneity found and the lack of complete data regarding the selected clinical outcomes, a comparative analysis has been possible by the categorization of used biomaterials and surgical flap approaches. This method led us to observe the best performance of grafts with the use of adjunctive PRP in CAL gain and PPD reduction. No difference has been outlined with a specific surgical flap.

**Conclusions:**

Although PRP is considered a cheap and patient's derived growth factor, the not conclusive data reported would suggest that its use in addition to bone substitutes could be of some clinical benefit in the regenerative treatment of infrabony defects.

**Clinical Relevance:**

This systematic review was intended to sort out the huge controversial debate in the field about the possible use of PRP in regenerative surgery in infrabony defect. The clinical relevance of using blood-borne growth factors to conventional procedures is effective as these could determine a better performance and outcomes despite the surgical approach adopted and limit the use of additional biomaterials for the blood clot stabilization.

## 1. Introduction

The ultimate goal of periodontal therapy in the case of infrabony defects is regeneration. Regeneration means “reproduction or reconstitution of a lost or injured part. It takes into account all the procedures attempting to regenerate lost periodontal structures through differential tissue responses and by different biomaterials such as grafts, membranes or Biomodulators as Enamel Matrix Proteins” (*Glossary of Periodontal Terms*, AAP, 2001). These procedures would temporarily delay the apical migration of the gingival epithelium allowing the granulation tissue derived from the periodontal ligament and osseous tissue to repopulate the space adjacent to the denuded root surface [1].

Several materials are available in daily practice, but none can be considered as an ideal one. In order to achieve a successful regeneration, biomaterials should fulfil four main characteristics: (1) structural integrity, (2) to work as a scaffold for tissue ingrowth, (3) favoured by stem cells that can potentially differentiate and support the regeneration, and (4) contain factors for regeneration and/or repair.

Growth factors (GFs) are expressed during different phases of healing, and they are key elements in promoting regeneration of tissues; these are considered the most relevant factors in osseoregenerative process.

Platelet-rich plasma (PRP) is considered a cheap way to obtain many growth factors (GFs) in physiological proportion, and it has already been largely applied as a GF's carrier in different tissues due to its properties of inducing healing response even in cases with low potential [2].

Substantially, PRP is a blood derivate growth factor with a higher platelet concentration up to 338% that can release all polypeptide GFs from alpha granules: platelet-derived growth factor (PDGF), transforming growth factor-*β*1 and transforming growth factor-*β*2 (TGF-*β*1 and TGF-*β*2), and insulin-like growth factors 1 and 2 (IGF-1,2) [3].

In clinical dental practice, the effective use of PRP has been described in sinus grafting procedures [4], alveolar socket preservation techniques [5], and also as an adjunctive procedure to support the regenerative process in periodontal infrabony and furcation defects [6].

Although its clinical benefits have been demonstrated several times, the adjunctive use of autologous PRP in regenerative procedures has produced controversial outcomes ranging from significant to null effects, as demonstrated from different published systematic reviews [3, 7].

This review investigates and updates the clinical efficacy of PRP when added to grafting materials and/or to membranes or biomodulators in cases of periodontal infrabony defects in patients with advanced chronic periodontitis. In particular, it was aimed to highlight the most relevant clinical outcome changes (vertical pocket probing depth, vertical clinical attachment level, and the recession) in GTR coupled with PRP compared to the same procedure without it. In order to get stronger evidence, a SR on published RCTs was chosen.

## 2. Materials and Methods

### 2.1. Focused Question

The focused question that this systematic review is intending to answer is

“What are the Vertical Probing Pocket Depth Reductions, the Vertical Clinical Attachment Level Gains and the Recession Reduction at infra-bony defects at least 6 months after Regenerative Surgery with the adjunctive use of PRP as documented in Randomized Clinical Trials, compared to the same clinical procedures and biomaterials performed without the use of PRP?”

### 2.2. Eligibility Criteria for Studies to be Included in This Review

According to the P.I.C.O system [8], inclusion criteria were outlined as follows:

#### 2.2.1. Study Population

Studies were limited to human subjects older than 18 years and in good general health, with a diagnosis of chronic periodontitis and with at least one pair of specular infrabony defects. Studies considering individuals with a history of aggressive periodontitis or conducted on animal models were excluded from our consideration.

#### 2.2.2. Type of Interventions

GTR surgical procedures with and without PRP will be the interventions considered for the comparative evaluation. The specific regenerative techniques and biomaterials investigated in this study were

1. autologous bone grafts,
2. bone substitutes (allogenic, xenogenic, and synthetic grafts),
3. barrier membranes (resorbable and not resorbable),
4. enamel matrix proteins (EMD).

#### 2.2.3. Type of Comparison

Infrabony defects treated by regenerative surgery with PRP were considered the test group and compared to the same defects treated by the same regenerative therapy without PRP that were considered the control group.

#### 2.2.4. Outcome Measures

Outcome variables considered in this study were

- probing pocket depth reduction (PPDRed mm),
- clinical attachment level gain (CALGain mm),
- recession reduction (RECRed).

These were evaluated as the mean difference (mm) from the time of surgery until the end of the evaluation period not before 6 months.

#### 2.2.5. Types of Studies

To be considered for inclusion in this review, studies should be randomized controlled clinical trials (RCTs) only; no cohort studies (CHT) or case-control studies were included. Case series and case reports studies were also not considered as they would provide a low strength of evidence.

### 2.3. Information Sources

The search has been performed by the use of the following electronic databases: Pubmed, Cochrane Oral Library, Embase, and LILACS.

Trial registers have been searched using Current Controlled Trials (http://www.controlled-trials.com/), ClinicalTrials.gov (http://clinicalTrials.gov/ct2/home) and the World Health Organization International Trials Registry Platform search portal (http://www.who.int/trialsearch/Default.aspx).

Conference abstracts have been searched using the ISI Web of knowledge (http://isiwebofknowledge.com) and the Grey literature using Open Grey (http://www.opengrey.eu).

Hand searching included a complete search of Journal of Periodontology and Journal of Clinical Periodontology up to December 2016 and bibliographies of all relevant papers and review articles. In the case of ambiguous or missing data, experts have been contacted directly.

The search has been performed up to and including December 2016.

## 3. Search Strategy

The following search strategy has been used as a combination of MeSH terms and free text words:

- Intervention and materials: (“PRP” [txt words] OR “Platelet Rich Plasma” [txt words] OR “Platelet” [mesh] OR “guided tissue regeneration” [mesh] OR “periodontal” [all fields] OR “regeneration [mesh] OR “guided-tissue-regeneration” [txt words] OR “GTR” [txt words] OR “periodontal regeneration” [txt words] OR “Bone filler” [txt words] OR “Autologous bone grafts” [txt words] OR “Autogenous bone” [txt words] OR “bone substitutes” [mesh] OR “allogenic grafts” [txt words] OR “Allografts” [txt words] OR “xenogenic grafts” [txt words] OR “xenografts” [mesh] OR “synthetic grafts” [txt words] OR “Barrier membranes” [txt words] OR “membranes” [mesh] OR “resorbable membrane” [txt words] OR “non-resorbable membrane” [txt words] OR “guided bone regeneration” [txt words] OR “GBR” [txt words] OR “freeze dried bone allograft” [txt words] OR “demineralized freeze dried bone allograft” [txt words] OR “DFDBA” [txt words] OR “FDBA” [txt words] OR “Bio-Oss” [txt words] OR “Bio-Oss Collagen” [txt words] OR “Alloplast” [txt words] OR “tricalciumphosphate” [txt words] OR “cerasorb” [txt words] OR “Bioglass” [txt words] OR “polymeric” [txt words] OR “collagen sponge” [txt words] OR “Collagen” [txt words] OR “Biogide” [txt words] OR “Ossix” [txt words] OR “Gore tex” [txt words] OR “Enamel Matrix Proteins” [txt words] OR “Emdogain” [txt words] OR “EMD” [txt words]);
- Disease: (“periodontal defects” [mesh] OR “periodontal [all fields] OR “infrabony defects” [txt words]);
- Study design: (.”randomized clinical trials” [mesh] OR randomized controlled study” [mesh] OR “clinical trial” [mesh] OR “cohort study” [mesh] OR “clinical trial” [mesh] OR “comparative study” [mesh] OR “systematic review” [mesh] OR “case control study” [mesh] OR “longitudinal study” [mesh]).

Adopted filters have been “humans,” and articles were published in English language.

### 3.1. Methods of the Review

#### 3.1.1. Screening and Selection

Initially, titles and abstracts of all reports were screened independently by two reviewers (MS and FP). Subsequently, for studies appearing to meet the inclusion criteria, or for which there were insufficient data in the title and abstract to make a clear decision, the full report was obtained and independently assessed by three reviewers (MS, FP, and MA) to establish whether the study met the inclusion criteria. Any disagreements were resolved by discussion among the reviewers. All studies meeting the inclusion criteria then underwent validity assessment. The reasons for rejecting studies at this or at subsequent stages were recorded.

Special attention was paid not to duplicate publications in order to avoid a likely bigger impact of the same data in the global result.

#### 3.1.2. Quality Assessment/Risk of Bias

The quality assessment of the included studies was undertaken independently by two reviewers based on the content of the articles. The reviewers were blind to the name of the authors, institutions, and journal titles.

A commonly used three-item, five-point quality scale was used to rate the quality of the studies [9]. The minimum score for the inclusion was 2, and the maximum was 5.

Points were awarded according to the following criteria:

1. Was the study randomized? If yes, +1 point.

Was the randomization procedure appropriate and clearly reported in the study? If yes, +1 point. If no, all points deleted.

(2) Was the study double-blinded? If yes, +1 point.

Was the double-blinding method appropriate and clearly reported in the study? If yes, +1 point. If no, all points deleted.

(3) Were the reasons for patient withdrawals and dropouts described, for each treatment group? If yes, +1 point.

A separate scoring for quality assessment was obtained and independently assessed by two reviewers (MS, FP) to establish whether the study met the quality criteria in order to reduce the risk of bias. The level of agreement between the two reviewers was calculated using kappa statistics.

#### 3.1.3. Data Extraction

Two reviewers (MS and FP) independently using specially designed data extraction forms extracted the necessary data. Any disagreement was discussed, and a third reviewer (MA) was consulted when necessary.

Authors of studies were contacted for clarification or missing information. Data was excluded until further clarification could be available or if an agreement could not be reached. When the results of a study were published more than once or results were detailed in a number of publications, the most complete data set was sought from all sources and included only once.

Using a standard protocol, the following data were collected from the studies:

- name of the authors, date of publication, name of the journal, and setting;
- details on the study design;
- sample size (number/gender);
- follow-up (months);
- treated infrabony defects (number), position of the defects (maxillary/mandibular);
- intervention/barrier-augmentation material, soft tissue closure, eventual antibiotic intake, reassessment;
- control group: intervention/barrier-augmentation material;
- clinical attachment level gain (CALgain);
- pocket probing depth reduction (PPDRed);
- recession reduction (RECRed).

#### 3.1.4. Heterogeneity Assessment

The statistical heterogeneity among studies has been assessed in two different ways: Cochran's Q statistical test [10] and *I*^2^ test [11] were applied to the selected studies. A fixed-effects model was adopted due to the hypothesis of a population of studies with similar characteristics.

In the case of high heterogeneity values, subgroups, and sensitivity, analysis was performed based on

- study site (maxillary/mandibular);
- regenerative material/s used (bone graft and/or resorbable/not resorbable barrier);
- surgical technique used.

#### 3.1.5. Data Synthesis

To summarize and compare studies, data were displayed as a weighted mean difference (WMD) in primary and secondary outcomes. Using this index, data from articles was directly pooled together (means and 95% CI).

- For dichotomous outcomes, the estimates were expressed as relative risk ratio (RR) together with 95% CI.
- For continuous outcomes, standardized mean differences and 95% confidence intervals were used to summarize the data for each study.

The study-specific estimates were pooled using the fixed-effects model (Woolf's method). If a significant heterogeneity was found, the random effect model result was presented.

Forest plots were created to illustrate the effects of the different studies and the global estimation.

SPSS Statistics™ software was used to perform all analyses. Statistical significance has been defined as a *p* value <0.05.

#### 3.1.6. Sensitivity Analysis and Bias Detection

Sensitivity analysis was performed excluding each of the studies step by step from the meta-analysis and evaluating the changes in the global estimation.

Publication biases were evaluated using a funnel plot and Egger's linear regression method.

#### 3.1.7. Final Recommendation

A final recommendation will be extracted from the results of this meta-analysis, considering their clinical significance.

## 4. Results

### 4.1. Study Selection

The search identified 39 articles on a record of 73 further filtered for “Humans,” “Clinical Trial,” and “English Language.” The independent screening of the titles and abstracts led to the rejection of 18 papers. The full text of the remaining 21 papers was then searched. For 1 study, the full text was not obtained [12], so the final pool was 20 studies. Out of these, 3 papers were further rejected for the following reasons: two studies did not provide a control group [13, 14] and one was not a fully RCT [15]. Two articles were rejected because they did not provide comprehensive data and/or standard deviations to be analysed [16, 17].

On the first screening, agreement between the reviewers was met for all the articles except one [18], because of the lab method for PRP gel preparation. The 3rd reviewer solved the debate by accepting it.

**Figure 1:**
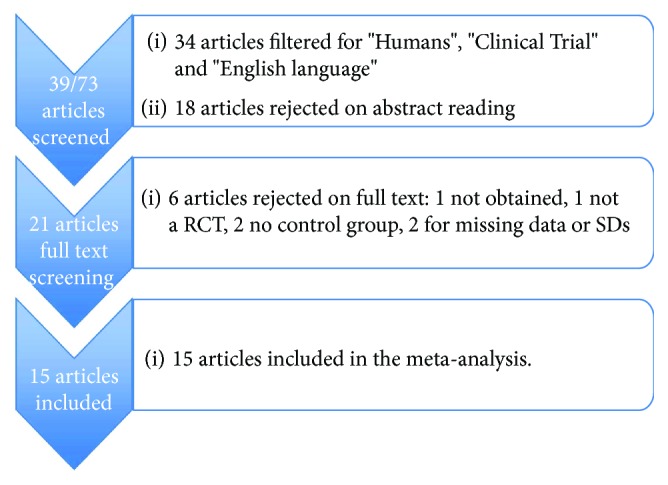
Flow chart of the screening process.

The final number of included studies was 15, and their characteristics are reported in Table 1. Not all the considered studies reported the mean change and the SD value for each outcome; in these cases, the studies were excluded from meta-analysis regarding the missing data (Figure 1).

#### 4.1.1. Classification of Studies according to Treatments

The included studies were grouped according to provided treatment. This action allowed us to analyse better the performance of PRP adding in the following test groups:

Group 1: biomodulators versus biomodulators and PRP (2 articles),

Group 2: grafts versus grafts and PRP (6 articles),

Group 3: none versus PRP alone (1 article),

Group 4: grafts and membranes versus grafts, membranes, and PRP (6 articles).

### 4.2. Methodological Quality of Included Studies

The quality of the included studies was assessed according to Jadad scoring [9].

Scoring was independently assessed by the reviewers, and all the studies reported a minimum of 2 points or above, allowing them to be included.

To test the extent of interagreement between the two reviewers, Cohen's Kappa Statistics was used.

Its value lies between −1 and 1, where 1 is the perfect agreement, 0 is exactly what would be expected by chance, and negative values indicate agreement less than chance, that is, potential systematic disagreement.

The calculated point estimate of Cohen's kappa statistic *κ* was 0.74, which according to the commonly cited scale for interpretation of kappa statistic (Landis and Koch [33]) indicates a substantial agreement between the two reviewers. The *Z* score = 5.77 with *p* value <.0001 showed that *κ* is different from zero. The 95% confidence limits for *κ* were (0.52, 0.97).

### 4.3. Heterogeneity Assessment

In order to evaluate if a within-study or between-study variability occurred, heterogeneity was assessed. Cochran's Q test was calculated although the small number of included studies led to the consideration of *I*^2^ statistics in a fixed-effects model. The *I*^2^ statistics showed a substantial heterogeneity for VCAL and VPD outcomes, while no heterogeneity was found according to REC.

### 4.4. Change in Vertical Clinical Attachment Level (VCAL) (Closed Assessment)

**Figure 2:**
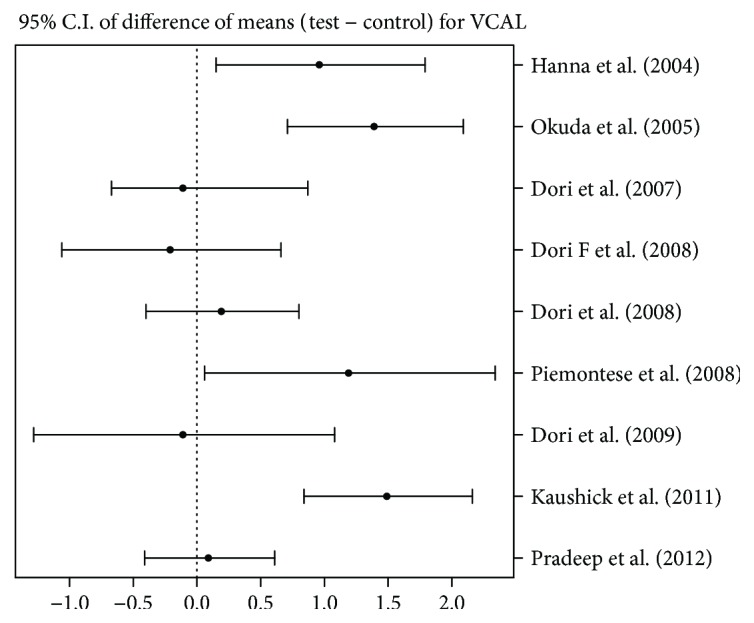
95% confidence interval presentation for difference between means of test group and control group for VCAL, that is, mean (test) − mean (control).

To test the effectiveness of using PRP in addition to the adopted treatment, 95% confidence intervals were constructed for difference between the means of test and control groups. The graphical presentation is reported in Figure 2. Out of nine articles, results of five articles led us to accept the hypothesis of no difference between the test and control groups, whereas four articles (Hanna et al. [31], Okuda et al. [29], Piemontese et al. [25], and Kaushick et al. [22]) suggested results in favour of the test group as the SDs showed a mean CAL gain of 2 mm compared to the control group.

### 4.5. Change in Vertical Probing Depth (VPD)

Twelve articles were able to provide data regarding vertical probing depth (VPD).

**Figure 3:**
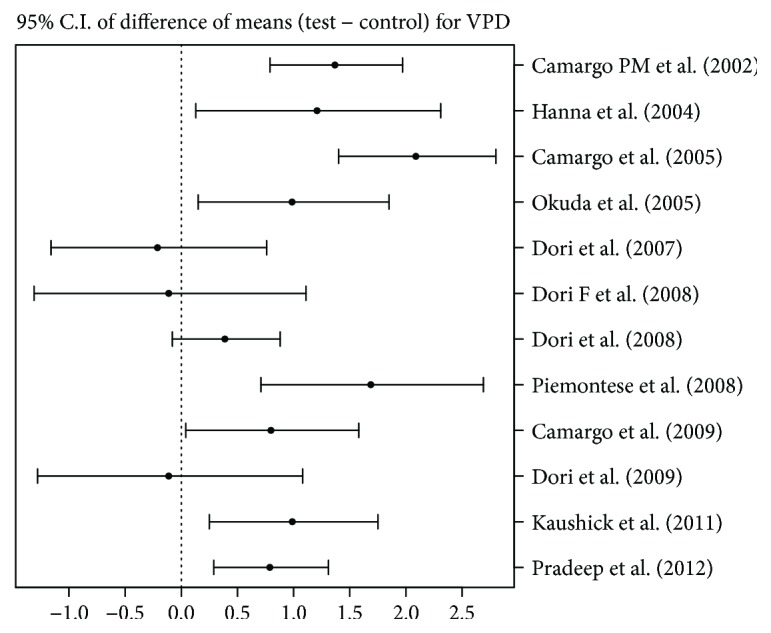
95% confidence interval presentation for difference between means of test group and control group for VPD, that is, mean (test) − mean (control).

Four out of these 12 [23, 26–28] studies showed no difference between the test and control groups, whereas the remaining 8 favoured the addition of PRP showing a VPDRed of about 1.5 mm (see Figure 3).

### 4.6. Change in Recession

**Figure 4:**
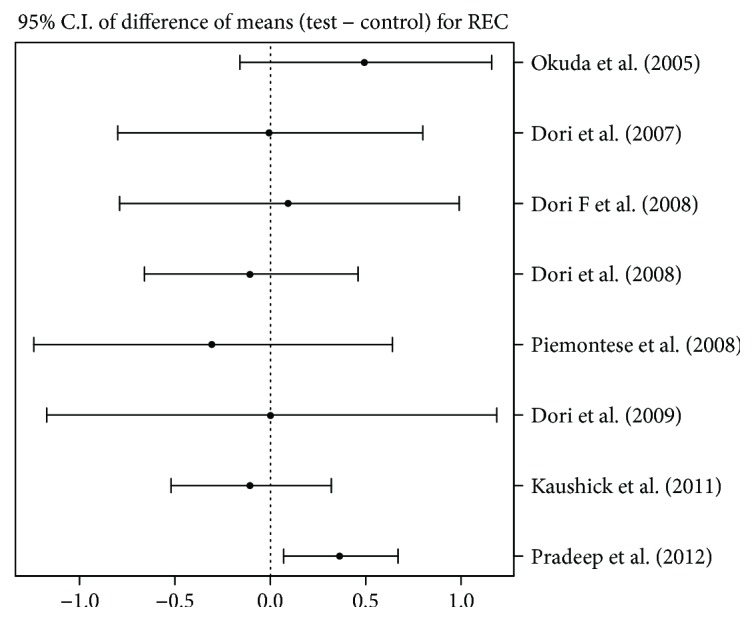
95% confidence interval presentation for difference between means of test group and control group for REC, that is, mean (test) − mean (control).

Out of 8 articles providing useful data for analysis, only one [20] seemed to show the effectiveness of the test group versus the control group in recession reduction of about 0.5 mm after treatment. The other seven studies did not provide any evaluable difference between groups (see Figure 4).

### 4.7. Change in Clinical Outcomes Regarding Treatment Groups

According to the categorization of treatments into 4 groups, only 2 of them provided evaluable data regarding the adopted clinical outcomes: grafts + PRP and grafts + PRP + membranes.

Biomodulators and PRP alone included one single evaluable observation as the other selected articles did not provide any mean baseline-final VCAL, VPD, and REC change or the SD.

When comparing VPD in grafts + PRP and grafts + PRP + membrane, five articles' sample data were available for each of X ¯grafts + PRP and X ¯grafts + PRP + membrane. The *t* statistic value *t* = 4.60 with *p* value <0.0001 suggested that we may reject null hypothesis in favour of *μ*grafts + PRP at *α* = 5%.

When comparing VCAL in “grafts + PRP” and “grafts + PRP + membrane,” two articles' sample data were available for X ¯grafts + PRP and five articles' data for X ¯grafts + PRP + membrane. The *t* statistic value *t* = 2.86 with *p* value = 0.0045 suggested that we may reject null hypothesis in favour of *μ*grafts + PRP at *α* = 5%.

When comparing REC in “grafts + PRP” and “Grafts + PRP + membrane,” two articles' data were available for computing X ¯grafts + PRP whereas four available articles provided values for X ¯grafts + PRP + membrane. The *t* statistic value *t* = 8.68 with *p* value <0.0001 again suggested to reject null hypothesis in favour of *μ*grafts + PRP at *α* = 5%.

### 4.8. Comparison of Clinical Outcomes Regarding the Adopted Surgical Technique

For VCAL, the mean of the test group, that is, “coronally placed” (based on test group of seven articles) was compared with the mean of control group, that is, “original position” (based on test group of two articles).

For VPD, the mean of the test group, that is, “coronally placed” (based on test group of seven articles) is compared with the mean of control group, that is, “original position” (based on test group of five articles).

For VCAL, to test the hypothesis H0, *μ*coronally placed = *μ*original position, X ¯coronally is computed on the basis of seven observations/articles and X ¯original is the mean of two observations/articles. The value of *t* statistic is calculated as *t* = 0.16 with *p* value = 0.3777. So we may accept the null hypothesis of equality of two means at 0.05 level of significance.

For VPD, to test the hypothesis H0, *μ*coronally placed = *μ*original position, X ¯coronally is computed on the basis of seven observations/articles and X ¯original is the mean of five observations/articles. The value of *t* statistic *t* = −1.26 with *p* value = 0.2071 again leads to accept the equality of two means at 0.05 level of significance.

## 5. Discussion

The present systematic review was intended to investigate the controversial results raised from similar papers already published and to update those. The objective was to provide a possible evidence for a better performance of regenerative surgery in infrabony defects with the adjunctive use of autologous blood-derived growth factors as PRP or focusing on the different surgical techniques adopted and subsequently to address future research on the topic.

According to the CONSORT guidelines, our consideration aimed to include only RCTs with a quality assessment equal or more than 2 according to Jadad classification. This allowed us to include only 15 articles from a starting batch of 73.

Although we followed a strict selection and the quality testing of the included studies, a significant heterogeneity was found, leading us to implement other strategies for categorizations of studies in order to assess and solve it. The adopted solutions categorized the studies according to the treatment in 4 classes (biomodulators, grafts, grafts + resorbable membranes, and treatment without materials). It also categorized it according to the surgical flap approach (coronally placed or replaced).

Another problem was the high number of the studies that did not provide standard deviations about the provided outcome values. This matter did not allow us to consider them in the meta-analysis due to the need of data regarding the sample variations and of the mean change of the outcomes from baseline examination to the reassessment after the follow-up.

### 5.1. Overall Intergroup Analysis

The overall meta-analysis performed on the main clinical outcomes (VPD, VCAL, and REC) led us to explore the behaviour of the test group (PRP added) in favouring a better healing in infrabony defect.

The reported 95% confidence intervals of 4 main studies [22, 25, 29, 31] were presenting favourable results for VPD or VCAL, while not the same for REC. This aspect would suggest an efficient clinical attachment gain inside the infrabony defect due to the adjunctive use of PRP as an appropriate regenerative method. Nevertheless, no radiographic comparison of the alveolar bone levels before and after treatment has been reported or systematically assessed, so we cannot provide a precise interpretation about the healing process.

In all the considered studies, the main treatment performed was the combination of a bone graft (HA, DFDBA, and TCP) with PRP without the use of any barrier or a membrane. The performed surgical procedure was flap repositioning without any coronal advancement.

### 5.2. Intragroup Analysis

When comparing the efficacy of different regenerative materials or techniques among them, the analysis was possible only when considering 2 out of 4 classes of categorization due to missing data.

The combination of bone grafts with PRP was always producing better clinical results in terms of CALgain and pocket reduction than the adjunctive use of membranes after short- and longer term reassessment.

This finding is in line with another systematic review on the same topic [7], which suggested that maybe PRP itself can act as a barrier due to the dense fibrin network produced after platelet activation. The adjunctive use of a membrane either resorbable or not could not allow any interaction between the chemokines and GFs released in the wound area and the overlying connective tissue.

When we looked if the grafting material showed any possible effect, no differences have been identified favouring a specific category. It is therefore suggested that the grafting material is acting as a scaffold leaving the PRP to execute the inductive phase of the healing process.

### 5.3. Adopted Surgical Technique

According to the evidence of a better performance in CALgain of coronally placed flaps in the regenerative approach to infrabony defects [34], our investigation moved to analyse if the adopted surgical technique or the use of adjunctive PRP could justify the observed better results in the test groups of selected studies. The considered surgical approaches were the replacement and the coronal placement. Due to the assessed high heterogeneity, a categorization according to the technique was performed using the test groups (PRP added) of the included studies. In comparison, the test group was the coronally advanced flap and the control was the replaced flap.

Although not the same number of articles were reporting the use of each one, a “*t* test” was possible considering only 2 main clinical outcomes: VPD and VCAL.

In all cases, no differences in terms of CAL gain or PPD reduction between the two adopted techniques have been highlighted. The blood clot stability was achieved in both conditions, and the healing process could reasonably happen due to the presence of the growth factors. Even the recession reduction could be outlined with the replaced flap as the evidence in the overall analysis regarding REC outcomes has been suggested [20].

## 6. Conclusions

We conclude from the data in this systematic review that the adjunctive use of PRP in the regenerative treatment of infrabony defects can be considered as an affordable technique to get a better CAL gain and PPD reduction in the surgical treatment of periodontal infrabony defects. Anyway, the limitations of the provided studies are the lack of baseline data regarding the defect size and their morphology, the absence of reports of other relevant clinical outcomes, as the bone fill, and the heterogeneity between studies.

On the basis of this systematic review, the regeneration/repair of infrabony defects would favour the use of adding PRP to a simple surgical repositioned flap technique, like in the open flap debridement (OFD), with the use of bone grafts (xenografts, HA, or TCP). No better results would be achievable using combinations with biomodulators (Emdogain) or membranes, the PRP just would act as a biomodulator itself.

In a biological sense, this observation would state for the biomolecular signalling action between PRP and the surrounding cellular environment that any membrane could interrupt or modify. The use of bone grafts would state as a blood clot stabilizer enhancing the osteoinductive properties of the PRP itself.

## 7. Future Research/Observations

According to the main reported pitfalls, future studies should be aimed first, designed according to RCT schemes in order to provide clinical evidences.

A comparison between a surgical flap approach alone and the adjunctive use of PRP would be needful in order to explore the role of growth factors alone in periodontal regeneration and the healing process, as well as the radiographic bone level assessment before and after treatment, as they represent a critical parameter in success assessment.

In order to explore which growth factor would be better suited in periodontal procedures, a multiple-arm RCT would be needful comparing PRP with other blood-derived agents available as well as with the different techniques adopted to deliver it.

## Figures and Tables

**Table 1:** Included studies.

Study	Design	Study location	Population mean age	M/F ratio	Intervention	Outcomes	Follow-up	Flap placement
[19]	RCT BA	Department of Periodontology, Semmelweis University, Budapest	32–56 years	13 M/11 F	EMD + NBM + PRP versus EMD + NBM	Regenerative surgery of deep infrabony defects	12 months, 5 years	Coronal advancement
[18]	RCT SM	University of Dammam, College of Dentistry, Kingdom of Saudi Arabia	41.4 ± 2.61 years	7 M/5 F	PRP + torus mandibularis chips versus torus mandibularis chips	Treatment for periodontal infrabony defects	6 months	Original
[20]	RCT	Department of Periodontics, Government Dental College and Research Institute, Bangalore, India	36.8 years	NA	PRP versus OFD	Treatment for periodontal infrabony defects	9 months	Coronal advancement
[21]	RCT	Department of Periodontology, Faculty of Dentistry, Gazi University, Ankara, Turkey	48.96 ± 6.6 years	9 M/5 F	B-TCP + PRP versus B-TCP	Therapeutic efficacy of platelet-rich plasma in infrabony defects	6 months	Original
[22]	RCT SM	Department of Periodontia, Saveetha Dental College and Hospitals Chennai	20 and 50 years	NA	PRP + HA + B-TCP versus HA + TCP	Clinical effectiveness of regenerative techniques	6 months	Original
[23]	RCT BA	Department of Periodontology, Semmelweis University, Budapest, Hungary	28 to 65 years	9 M/21 F	PRP + ABBM versus ABBM	Modality to enhance the outcome of regenerative surgery	12 months	Coronal advancement
[24]	RCT SM	School of Dentistry, University of Belgrade, Republic of Serbia	47 ± 10 years	9 M/14 F	BPBM + GTR + PRP versus BPBM + GTR	Additional benefits provided by the incorporation of platelet-rich plasma (PRP) into a regenerative protocol	6 months	Original
[25]	RCT	Division of Periodontology, Polytechnic University of Marche, Ancona Torrette, Italy	47 to 72 years	31 M/29 F	DFDBA + PRP versus DFDBA	Compare PRP combined with DFDBA to DFDBA in the treatment of human intrabony defects	12 months	Coronal advancement
[26]	RCT BA	Department of Periodontology, Semmelweis University, Budapest, Hungary	28 to 58 years	12 M/16 F	PRP + B-TCP + GTR versus B-TCP + GTR	To enhance the outcome of regenerative surgery	12 months	Coronal advancement
[27]	RCT	Department of Periodontology, Semmelweis University, Budapest, Hungary	32–56 years	12 M/14 F	EMD + NBM + PRP versus EMD + NBM	To enhance the outcomes of regenerative surgery significantly	12 months	Coronal advancement
[28]	RCT BA	Department of Periodontology, Semmelweis University, Budapest, Hungary	26 to 55 years	10 M/14 F	PRP + ABBM + GTR or ABBM + GTR	To clinically evaluate the effect of PRP on the healing of deep intrabony defects	12 months	Coronal advancement
[29]	RCT	Division of Periodontology, Niigata University Graduate School of Medical and Dental Sciences, Niigata, Japan	55.5 ± 8.2 years	21 M/49 F	PRP + HA versus HA + saline	To compare platelet-rich plasma (PRP) combined with a biodegradable ceramic in the treatment of human intrabony defects	12 months	Original
[30]	RCT SM	School of Dentistry, University of California, Los Angeles	41 ± 13 years	16 M/12 F	BPBM + GTR + PRP versus OFD	Clinical effectiveness of a combination therapy in the regeneration of periodontal intrabony defects in humans	6 months	Original
[31]	RCT SM	Private practice, Houston and Department of Periodontics, The University of Texas Health Science Center at Houston, TX	37 to 74 years	5 M/8 F	PRP + BDX versus BDX	The combination of PRP and BDX to those obtained from the use of the bone replacement graft alone	6 months	Coronal advancement
[32]	RCT SM	Clinical Specialties, Section of Periodontics, UCLA School of Dentistry, Los Angeles, CA, USA	39 ± 9 years	8 M/10 F	PRP + BPBM + GTR versus GTR	Combination therapy in promoting clinical signs of periodontal regeneration in intrabony defects	6 months	Original

EMD: enamel matrix derivative; NBM: natural bone mineral; PRP: platelet-rich plasma; OFD: open flap debridement; B-TCP: beta-tricalcium phosphate; ABBM: anorganic bovine bone mineral; BPBM: bovine porous bone mineral; GTR: guided tissue regeneration; DFDBA: demineralized freeze-dried bone allograft; BDX: bovine-derived xenograft.
